# Bis(tripyrazol-1-ylmethane)nickel(II) tetra­cyanidonickelate(II) dihydrate

**DOI:** 10.1107/S1600536809046108

**Published:** 2009-11-07

**Authors:** Ganna Lyubartseva, Sean Parkin

**Affiliations:** a Department of Biology and Chemistry, College of Science and Technology, Southern Arkansas University, Magnolia AR 71753, USA; b Department of Chemistry, University of Kentucky, Lexington KY 40506, USA

## Abstract

The title complex, [Ni(C_10_H_10_N_6_)_2_][Ni(CN)_4_]·2H_2_O, contains an octa­hedral nickel(II) cation and a square-planar nickel(II) anion. Both the cation and the anion reside on a crystallographic center of inversion. The Ni^II^ center in the cation is coordinated by six pyrazol-1-yl rings of two chelating tripyrazol-1-ylmethane [HC(pz)_3_] ligands, with Ni—N distances that range between 2.0647 (19) and 2.0828 (19) Å. The Ni^II^ center in the anion is coordinated by four cyanide ligands, with Ni—C distances in the range 1.869 (2)–1.869 (3) Å. The [Ni(CN)_4_]^2−^ anions are linked by inversion-related water mol­ecules into extended chains that run parallel to the *a* axis.

## Related literature

For the ligand synthesis, see: Reger *et al.* (2000 ▶). For allowed and forbidden *d*–*d* transitions in poly(3,5-dimethyl­pyrazol­yl)methane complexes of nickel(II), see: Nolet *et al.* (2006 ▶). For coupled electron-transfer and spin-exchange reactions of metal-bis­[tris­(pyrazol­yl)methane] complexes, see: Sheets & Schultz (2004 ▶). For structural, spectroscopic and angular-overlap studies of tris­(pyrazol-1-yl)methane complexes, see: Astley *et al.* (1993 ▶). For nickel(II) complexes of some poly(1-pyrazol­yl)alkane ligands, see: Mesubi & Ekemenzie (1984 ▶). For the coordination chemistry of geminal poly(1-pyrazol­yl)alkanes, see: Trofimenko *et al.* (1970 ▶). 
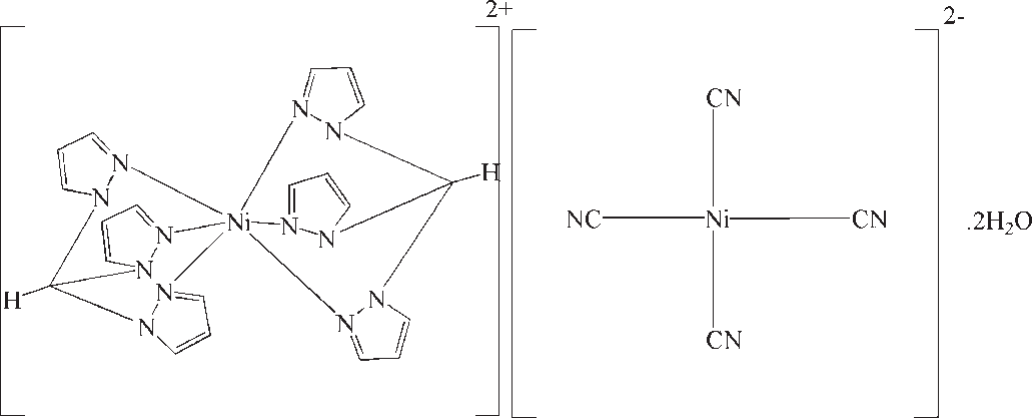


## Experimental

### 

#### Crystal data

[Ni(C_10_H_10_N_6_)_2_][Ni(CN)_4_]·2H_2_O
*M* *_r_* = 686.01
Triclinic, 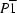

*a* = 8.4840 (2) Å
*b* = 8.7355 (2) Å
*c* = 10.7522 (3) Å
α = 75.5129 (11)°
β = 75.2713 (12)°
γ = 89.2534 (12)°
*V* = 745.09 (3) Å^3^
*Z* = 1
Mo *K*α radiation
μ = 1.32 mm^−1^
*T* = 90 K
0.24 × 0.21 × 0.20 mm

#### Data collection

Nonius KappaCCD diffractometer
Absorption correction: multi-scan (*SCALEPACK*; Otwinowski & Minor, 1997 ▶) *T*_min_ = 0.743, *T*_max_ = 0.779
13964 measured reflections
3404 independent reflections
2664 reflections with *I* > 2σ(*I*)
*R*_int_ = 0.063

#### Refinement

*R*[*F* ^2^ > 2σ(*F* ^2^)] = 0.038
*wR*(*F* ^2^) = 0.108
*S* = 1.08
3404 reflections
209 parameters
3 restraints
H atoms treated by a mixture of independent and constrained refinement
Δρ_max_ = 0.70 e Å^−3^
Δρ_min_ = −0.80 e Å^−3^

### 

Data collection: *COLLECT* (Nonius, 1998 ▶); cell refinement: *SCALEPACK* (Otwinowski & Minor, 1997 ▶); data reduction: *DENZO-SMN* (Otwinowski & Minor, 1997 ▶); program(s) used to solve structure: *SHELXS97* (Sheldrick, 2008 ▶); program(s) used to refine structure: *SHELXL97* (Sheldrick, 2008 ▶); molecular graphics: *XP* in *SHELXTL* (Sheldrick, 2008 ▶); software used to prepare material for publication: *SHELXL97* and local procedures.

## Supplementary Material

Crystal structure: contains datablocks global, I. DOI: 10.1107/S1600536809046108/om2294sup1.cif

Structure factors: contains datablocks I. DOI: 10.1107/S1600536809046108/om2294Isup2.hkl

Additional supplementary materials:  crystallographic information; 3D view; checkCIF report

## Figures and Tables

**Table 1:** Hydrogen-bond geometry (Å, °)

*D*—H⋯*A*	*D*—H	H⋯*A*	*D*⋯*A*	*D*—H⋯*A*
O1*W*—H1*W*⋯N8	0.83 (2)	1.97 (2)	2.798 (3)	176 (3)
O1*W*—H2*W*⋯N7^i^	0.83 (2)	1.99 (2)	2.815 (3)	174 (3)

Symmetry code: (i) 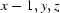.

**Fig. 1.**
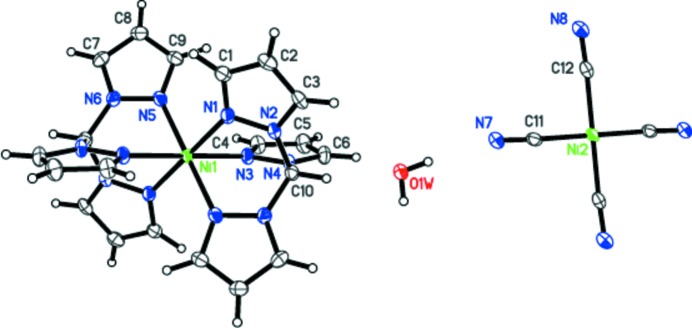
View of the title compound with the atom numbering scheme. Displacement ellipsoids are drawn at the 50% probability level.

## supplementary crystallographic information

### Comment

Tris(pyrazolyl)methane ligands, which are isoelectronic to the poly(pyrazolyl)borate ligand, are well known for their modulating ability towards magnetic, spectroscopic and catalytic properties of transition metal compounds (Nolet *et al.* (2006), Sheets *et al.* (2004), Astley *et al.* (1993), by Mesubi *et al.* (1984), Trofimenko *et al.* (1970)). They are also relatively easy to synthesize. During our search for a better catalyst for a large range of chemical reactions, we found tris(polypyrazolyl)methane ligand very promising. Here we report a new nickel (II) complex with this ligand as the tetracyanonickelate salt.

The title complex, [Ni(HC(pz)~3~)~2~][Ni(CN)~4~].2H~2Õ, where HC(pz)~3~ is tris(1-pyrazolyl)methane, contains an octahedral nickel(II) cation and a square planar nickel(II) anion (Fig. 1). Both the cation and the anion reside on a crystallographic center of inversion.The nickel atom in the cation is coordinated by six pyrazolyl rings of two chelating HC(pz)~3~ ligands, with Ni—N distances that range between 2.0647 (19) Å and 2.0828 (19) Å. The nickel atom in the anion part is coordinated by four cyanide ligands, with Ni—C distances in the range 1.869 (2) Å to 1.869 (3) Å. The Ni(CN)_4_^2-^ anions are linked by inversion-related water molecules into extended chains that run parallel to the *a* axis.

### Experimental

Tris(pyrazolyl)methane ligand was synthesized according to the previously published procedure by Reger *et al.* (2000). Tetraethyl ammonium cyanide was purchased from Aldrich and used as received. NiCl~2~.6H~2~0(475 mg, 2 mmol) was dissolved in 5 ml methanol. Tris(pyrazolyl)methane (428 mg, 2 mmol) was dissolved in 5 ml methanol. The ligand solution was added dropwise to metal solution and with moderate stirring. Once the addition was complete, solid tetraethylammonium cyanide (938 mg, 6 mmol) was added. The clear solution was filtered and methanol was evaporated slowly. Pink crystals were obtained after 3 days (463 mg, 67% yield). Elemental analysis, calculated for Ni~2~C~24~H~24Ñ~16Õ~2~: C 42.02, H 3.53, N 32.67; found C 41.95, H 3.30, N 32.45. IR (cm^-1^): 3330, 3279, 3145, 3110, 2986, 2132, 1687, 1516, 1450,1441, 1402, 1285, 1252, 1219, 1088, 1057, 987, 859, 791, 770, 658, 607, 414.

### Refinement

H atoms were found in difference Fourier maps and those attached to carbon were subsequently placed in idealized positions with constrained distances of 0.95 Å (C_Ar_H), and *U*_iso_(H) values set to 1.2*U*_eq_ (C). Water H atoms were refined subject to three geometry retraints (*DFIX* for the O- –H and H—H distances in *SHELXL97*) with *U*_iso_ set to 1.5*U*_eq_ (O_water_).

### Crystal data

**Table:**

[Ni(C_10_H_10_N_6_)_2_][Ni(CN)_4_]·2H_2_O	*Z* = 1
*M**_r_* = 686.01	*F*(000) = 352
Triclinic, *P*1	*D*_x_ = 1.529 Mg m^−^^3^
Hall symbol: -P 1	Mo *K*α radiation, λ = 0.71073 Å
*a* = 8.4840 (2) Å	Cell parameters from 3304 reflections
*b* = 8.7355 (2) Å	θ = 1.0–27.5°
*c* = 10.7522 (3) Å	µ = 1.32 mm^−^^1^
α = 75.5129 (11)°	*T* = 90 K
β = 75.2713 (12)°	Block, pink
γ = 89.2534 (12)°	0.24 × 0.21 × 0.20 mm
*V* = 745.09 (3) Å^3^	

### Data collection

**Table:**

Nonius KappaCCD diffractometer	3404 independent reflections
Radiation source: fine-focus sealed tube	2664 reflections with *I* > 2σ(*I*)
graphite	*R*_int_ = 0.063
Detector resolution: 9.1 pixels mm^-1^	θ_max_ = 27.5°, θ_min_ = 2.0°
ω scans at fixed χ = 55°	*h* = −10→11
Absorption correction: multi-scan (*SCALEPACK*; Otwinowski & Minor, 1997)	*k* = −11→11
*T*_min_ = 0.743, *T*_max_ = 0.779	*l* = −13→13
13964 measured reflections	

### Refinement

**Table:**

Refinement on *F*^2^	Primary atom site location: structure-invariant direct methods
Least-squares matrix: full	Secondary atom site location: difference Fourier map
*R*[*F*^2^ > 2σ(*F*^2^)] = 0.038	Hydrogen site location: inferred from neighbouring sites
*wR*(*F*^2^) = 0.108	H atoms treated by a mixture of independent and constrained refinement
*S* = 1.08	*w* = 1/[σ^2^(*F*_o_^2^) + (0.0568*P*)^2^ + 0.2597*P*] where *P* = (*F*_o_^2^ + 2*F*_c_^2^)/3
3404 reflections	(Δ/σ)_max_ < 0.001
209 parameters	Δρ_max_ = 0.70 e Å^−^^3^
3 restraints	Δρ_min_ = −0.80 e Å^−^^3^

### Special details

**Table:**

Geometry. All e.s.d.'s (except the e.s.d. in the dihedral angle between two l.s. planes) are estimated using the full covariance matrix. The cell e.s.d.'s are taken into account individually in the estimation of e.s.d.'s in distances, angles and torsion angles; correlations between e.s.d.'s in cell parameters are only used when they are defined by crystal symmetry. An approximate (isotropic) treatment of cell e.s.d.'s is used for estimating e.s.d.'s involving l.s. planes.
Refinement. Refinement of *F*^2^ against ALL reflections. The weighted *R*-factor *wR* and goodness of fit *S* are based on *F*^2^, conventional *R*-factors *R* are based on *F*, with *F* set to zero for negative *F*^2^. The threshold expression of *F*^2^ > 2σ(*F*^2^) is used only for calculating *R*-factors(gt) *etc*. and is not relevant to the choice of reflections for refinement. *R*-factors based on *F*^2^ are statistically about twice as large as those based on *F*, and *R*- factors based on ALL data will be even larger.Three restraints (*DFIX* in *SHELXL97*) were used to ensure the geometry of the water molecule remained chemically reasonable.

### Fractional atomic coordinates and isotropic or equivalent isotropic displacement parameters (Å^2^)

**Table:**

	*x*	*y*	*z*	*U*_iso_*/*U*_eq_	
Ni1	0.5000	0.5000	1.0000	0.01475 (13)	
N1	0.7163 (2)	0.4988 (2)	0.85558 (19)	0.0177 (4)	
C1	0.8185 (3)	0.3933 (3)	0.8155 (2)	0.0218 (5)	
H1	0.8023	0.2821	0.8520	0.026*	
N2	0.7864 (2)	0.6416 (2)	0.77724 (18)	0.0165 (4)	
C2	0.9533 (3)	0.4679 (3)	0.7125 (2)	0.0236 (5)	
H2	1.0423	0.4185	0.6673	0.028*	
N3	0.6175 (2)	0.6834 (2)	1.03923 (19)	0.0191 (4)	
C3	0.9294 (3)	0.6262 (3)	0.6912 (2)	0.0204 (5)	
H3	0.9996	0.7095	0.6281	0.024*	
N4	0.7081 (2)	0.7950 (2)	0.93389 (19)	0.0179 (4)	
C4	0.6513 (3)	0.7139 (3)	1.1448 (2)	0.0231 (5)	
H4	0.6050	0.6554	1.2339	0.028*	
N5	0.5640 (2)	0.3277 (2)	1.14631 (19)	0.0193 (4)	
C5	0.7636 (3)	0.8429 (3)	1.1081 (3)	0.0272 (6)	
H5	0.8065	0.8873	1.1655	0.033*	
N6	0.4508 (2)	0.2078 (2)	1.21934 (19)	0.0184 (4)	
C6	0.7992 (3)	0.8923 (3)	0.9720 (3)	0.0237 (5)	
H6	0.8726	0.9775	0.9159	0.028*	
C7	0.5121 (3)	0.0982 (3)	1.3053 (2)	0.0214 (5)	
H7	0.4560	0.0050	1.3661	0.026*	
C8	0.6700 (3)	0.1472 (3)	1.2881 (2)	0.0233 (5)	
H8	0.7458	0.0954	1.3339	0.028*	
C9	0.6972 (3)	0.2894 (3)	1.1892 (2)	0.0214 (5)	
H9	0.7975	0.3510	1.1567	0.026*	
C10	0.7123 (3)	0.7855 (3)	0.7998 (2)	0.0177 (5)	
H10	0.7806	0.8779	0.7348	0.021*	
Ni2	0.5000	0.5000	0.5000	0.01604 (13)	
N7	0.7352 (2)	0.7733 (3)	0.4652 (2)	0.0219 (5)	
N8	0.2614 (3)	0.7476 (3)	0.4345 (2)	0.0258 (5)	
C11	0.6471 (3)	0.6668 (3)	0.4813 (2)	0.0193 (5)	
C12	0.3489 (3)	0.6500 (3)	0.4610 (2)	0.0190 (5)	
O1W	0.0369 (2)	0.9370 (2)	0.32515 (19)	0.0279 (4)	
H1W	0.101 (3)	0.877 (3)	0.358 (3)	0.042*	
H2W	−0.053 (3)	0.894 (3)	0.369 (3)	0.042*	

### Atomic displacement parameters (Å^2^)

**Table:**

	*U*^11^	*U*^22^	*U*^33^	*U*^12^	*U*^13^	*U*^23^
Ni1	0.0122 (2)	0.0154 (2)	0.0150 (2)	−0.00204 (16)	−0.00209 (16)	−0.00215 (16)
N1	0.0165 (10)	0.0172 (9)	0.0178 (10)	−0.0021 (8)	−0.0031 (8)	−0.0027 (8)
C1	0.0178 (12)	0.0212 (12)	0.0275 (13)	0.0020 (10)	−0.0064 (10)	−0.0076 (10)
N2	0.0144 (10)	0.0186 (10)	0.0156 (9)	−0.0024 (8)	−0.0028 (8)	−0.0037 (7)
C2	0.0162 (12)	0.0310 (14)	0.0243 (13)	0.0041 (10)	−0.0030 (10)	−0.0109 (11)
N3	0.0175 (10)	0.0191 (10)	0.0173 (10)	−0.0041 (8)	−0.0019 (8)	−0.0011 (8)
C3	0.0130 (12)	0.0304 (13)	0.0165 (11)	−0.0032 (10)	−0.0014 (9)	−0.0058 (10)
N4	0.0171 (10)	0.0172 (10)	0.0166 (10)	−0.0055 (8)	0.0003 (8)	−0.0040 (8)
C4	0.0246 (13)	0.0272 (13)	0.0172 (12)	−0.0033 (10)	−0.0030 (10)	−0.0075 (10)
N5	0.0145 (10)	0.0209 (10)	0.0193 (10)	−0.0014 (8)	−0.0023 (8)	−0.0017 (8)
C5	0.0284 (15)	0.0304 (14)	0.0254 (13)	−0.0048 (11)	−0.0066 (11)	−0.0120 (11)
N6	0.0187 (10)	0.0177 (10)	0.0167 (10)	−0.0013 (8)	−0.0029 (8)	−0.0022 (8)
C6	0.0232 (14)	0.0200 (12)	0.0270 (13)	−0.0057 (10)	−0.0039 (10)	−0.0065 (10)
C7	0.0282 (14)	0.0175 (11)	0.0170 (12)	0.0033 (10)	−0.0050 (10)	−0.0025 (9)
C8	0.0221 (13)	0.0233 (12)	0.0263 (13)	0.0059 (10)	−0.0089 (10)	−0.0070 (10)
C9	0.0167 (12)	0.0246 (12)	0.0245 (13)	0.0029 (10)	−0.0072 (10)	−0.0076 (10)
C10	0.0160 (12)	0.0187 (11)	0.0162 (11)	−0.0012 (9)	−0.0023 (9)	−0.0020 (9)
Ni2	0.0109 (2)	0.0195 (2)	0.0168 (2)	−0.00070 (16)	−0.00297 (16)	−0.00337 (16)
N7	0.0150 (10)	0.0243 (11)	0.0240 (11)	−0.0005 (9)	−0.0023 (8)	−0.0046 (9)
N8	0.0160 (11)	0.0288 (12)	0.0312 (12)	−0.0002 (9)	−0.0060 (9)	−0.0053 (9)
C11	0.0143 (12)	0.0240 (12)	0.0181 (12)	0.0038 (10)	−0.0053 (9)	−0.0019 (9)
C12	0.0137 (12)	0.0244 (12)	0.0180 (12)	−0.0038 (10)	−0.0026 (9)	−0.0051 (9)
O1W	0.0168 (9)	0.0253 (10)	0.0340 (11)	−0.0026 (8)	−0.0064 (8)	0.0060 (8)

### Geometric parameters (Å, °)

**Table:**

Ni1—N5	2.0647 (19)	N5—N6	1.367 (3)
Ni1—N5^i^	2.0647 (19)	C5—C6	1.372 (4)
Ni1—N3	2.081 (2)	C5—H5	0.9500
Ni1—N3^i^	2.081 (2)	N6—C7	1.353 (3)
Ni1—N1	2.0828 (19)	N6—C10^i^	1.448 (3)
Ni1—N1^i^	2.0828 (19)	C6—H6	0.9500
N1—C1	1.332 (3)	C7—C8	1.364 (4)
N1—N2	1.363 (3)	C7—H7	0.9500
C1—C2	1.405 (3)	C8—C9	1.396 (3)
C1—H1	0.9500	C8—H8	0.9500
N2—C3	1.354 (3)	C9—H9	0.9500
N2—C10	1.446 (3)	C10—N6^i^	1.448 (3)
C2—C3	1.365 (3)	C10—H10	1.0000
C2—H2	0.9500	Ni2—C12^ii^	1.869 (2)
N3—C4	1.326 (3)	Ni2—C12	1.869 (2)
N3—N4	1.360 (3)	Ni2—C11	1.869 (3)
C3—H3	0.9500	Ni2—C11^ii^	1.869 (3)
N4—C6	1.356 (3)	N7—C11	1.151 (3)
N4—C10	1.457 (3)	N8—C12	1.153 (3)
C4—C5	1.396 (4)	O1W—H1W	0.83 (2)
C4—H4	0.9500	O1W—H2W	0.83 (2)
N5—C9	1.334 (3)		
			
N5—Ni1—N5^i^	180.00 (10)	C5—C4—H4	124.3
N5—Ni1—N3	93.82 (8)	C9—N5—N6	104.52 (18)
N5^i^—Ni1—N3	86.18 (8)	C9—N5—Ni1	137.45 (17)
N5—Ni1—N3^i^	86.18 (8)	N6—N5—Ni1	117.85 (14)
N5^i^—Ni1—N3^i^	93.82 (8)	C6—C5—C4	105.8 (2)
N3—Ni1—N3^i^	179.999 (1)	C6—C5—H5	127.1
N5—Ni1—N1	94.89 (8)	C4—C5—H5	127.1
N5^i^—Ni1—N1	85.12 (8)	C7—N6—N5	111.63 (19)
N3—Ni1—N1	85.04 (8)	C7—N6—C10^i^	129.2 (2)
N3^i^—Ni1—N1	94.96 (8)	N5—N6—C10^i^	119.13 (18)
N5—Ni1—N1^i^	85.11 (8)	N4—C6—C5	106.1 (2)
N5^i^—Ni1—N1^i^	94.88 (8)	N4—C6—H6	127.0
N3—Ni1—N1^i^	94.96 (8)	C5—C6—H6	127.0
N3^i^—Ni1—N1^i^	85.04 (8)	N6—C7—C8	106.9 (2)
N1—Ni1—N1^i^	180.00 (8)	N6—C7—H7	126.6
C1—N1—N2	104.50 (19)	C8—C7—H7	126.6
C1—N1—Ni1	138.05 (17)	C7—C8—C9	105.7 (2)
N2—N1—Ni1	117.40 (14)	C7—C8—H8	127.1
N1—C1—C2	111.3 (2)	C9—C8—H8	127.1
N1—C1—H1	124.4	N5—C9—C8	111.2 (2)
C2—C1—H1	124.4	N5—C9—H9	124.4
C3—N2—N1	112.04 (19)	C8—C9—H9	124.4
C3—N2—C10	128.24 (19)	N2—C10—N6^i^	111.23 (19)
N1—N2—C10	119.54 (18)	N2—C10—N4	109.41 (18)
C3—C2—C1	105.5 (2)	N6^i^—C10—N4	110.33 (18)
C3—C2—H2	127.3	N2—C10—H10	108.6
C1—C2—H2	127.3	N6^i^—C10—H10	108.6
C4—N3—N4	104.74 (19)	N4—C10—H10	108.6
C4—N3—Ni1	136.46 (17)	C12^ii^—Ni2—C12	179.999 (1)
N4—N3—Ni1	117.88 (15)	C12^ii^—Ni2—C11	91.72 (10)
N2—C3—C2	106.7 (2)	C12—Ni2—C11	88.28 (10)
N2—C3—H3	126.6	C12^ii^—Ni2—C11^ii^	88.28 (10)
C2—C3—H3	126.6	C12—Ni2—C11^ii^	91.72 (10)
C6—N4—N3	112.08 (19)	C11—Ni2—C11^ii^	179.999 (1)
C6—N4—C10	128.5 (2)	N7—C11—Ni2	177.0 (2)
N3—N4—C10	118.97 (19)	N8—C12—Ni2	176.9 (2)
N3—C4—C5	111.3 (2)	H1W—O1W—H2W	103 (2)
N3—C4—H4	124.3		
			
N5—Ni1—N1—C1	39.3 (3)	Ni1—N3—C4—C5	167.67 (19)
N5^i^—Ni1—N1—C1	−140.7 (3)	N3—Ni1—N5—C9	−44.9 (3)
N3—Ni1—N1—C1	132.7 (3)	N3^i^—Ni1—N5—C9	135.1 (3)
N3^i^—Ni1—N1—C1	−47.3 (3)	N1—Ni1—N5—C9	40.5 (3)
N5—Ni1—N1—N2	−137.72 (16)	N1^i^—Ni1—N5—C9	−139.5 (3)
N5^i^—Ni1—N1—N2	42.28 (16)	N3—Ni1—N5—N6	140.99 (16)
N3—Ni1—N1—N2	−44.30 (16)	N3^i^—Ni1—N5—N6	−39.01 (16)
N3^i^—Ni1—N1—N2	135.70 (16)	N1—Ni1—N5—N6	−133.67 (16)
N2—N1—C1—C2	0.1 (3)	N1^i^—Ni1—N5—N6	46.33 (16)
Ni1—N1—C1—C2	−177.13 (18)	N3—C4—C5—C6	−0.1 (3)
C1—N1—N2—C3	−0.5 (3)	C9—N5—N6—C7	0.1 (3)
Ni1—N1—N2—C3	177.41 (15)	Ni1—N5—N6—C7	175.99 (15)
C1—N1—N2—C10	−176.0 (2)	C9—N5—N6—C10^i^	178.2 (2)
Ni1—N1—N2—C10	1.9 (3)	Ni1—N5—N6—C10^i^	−5.9 (3)
N1—C1—C2—C3	0.3 (3)	N3—N4—C6—C5	−1.0 (3)
N5—Ni1—N3—C4	−30.7 (3)	C10—N4—C6—C5	−173.0 (2)
N5^i^—Ni1—N3—C4	149.3 (3)	C4—C5—C6—N4	0.6 (3)
N1—Ni1—N3—C4	−125.3 (3)	N5—N6—C7—C8	−0.2 (3)
N1^i^—Ni1—N3—C4	54.7 (3)	C10^i^—N6—C7—C8	−178.1 (2)
N5—Ni1—N3—N4	136.29 (17)	N6—C7—C8—C9	0.2 (3)
N5^i^—Ni1—N3—N4	−43.71 (17)	N6—N5—C9—C8	0.0 (3)
N1—Ni1—N3—N4	41.71 (16)	Ni1—N5—C9—C8	−174.60 (18)
N1^i^—Ni1—N3—N4	−138.29 (16)	C7—C8—C9—N5	−0.1 (3)
N1—N2—C3—C2	0.7 (3)	C3—N2—C10—N6^i^	123.8 (2)
C10—N2—C3—C2	175.7 (2)	N1—N2—C10—N6^i^	−61.6 (3)
C1—C2—C3—N2	−0.6 (3)	C3—N2—C10—N4	−114.1 (2)
C4—N3—N4—C6	0.9 (3)	N1—N2—C10—N4	60.6 (3)
Ni1—N3—N4—C6	−169.89 (16)	C6—N4—C10—N2	107.9 (3)
C4—N3—N4—C10	173.8 (2)	N3—N4—C10—N2	−63.7 (3)
Ni1—N3—N4—C10	3.0 (3)	C6—N4—C10—N6^i^	−129.4 (2)
N4—N3—C4—C5	−0.5 (3)	N3—N4—C10—N6^i^	59.0 (3)

Symmetry codes: (i) −*x*+1, −*y*+1, −*z*+2; (ii) −*x*+1, −*y*+1, −*z*+1.

### Hydrogen-bond geometry (Å, °)

**Table:**

*D*—H···*A*	*D*—H	H···*A*	*D*···*A*	*D*—H···*A*
O1W—H1W···N8	0.83 (2)	1.97 (2)	2.798 (3)	176 (3)
O1W—H2W···N7^iii^	0.83 (2)	1.99 (2)	2.815 (3)	174 (3)

Symmetry codes: (iii) *x*−1, *y*, *z*.
